# Antibiotic Resistance Characteristics of *Pseudomonas aeruginosa* Isolated from Keratitis in Australia and India

**DOI:** 10.3390/antibiotics9090600

**Published:** 2020-09-14

**Authors:** Mahjabeen Khan, Fiona Stapleton, Stephen Summers, Scott A. Rice, Mark D. P. Willcox

**Affiliations:** 1 School of Optometry and Vision Science, UNSW, Sydney, NSW 2052, Australia; mahjabeen.khan@student.unsw.edu.au (M.K.); f.stapleton@unsw.edu.au (F.S.); 2 The Singapore Centre for Environment Life Sciences Engineering (SCELSE), The School of Biological Sciences, Nanyang Technological University, Singapore 639798, Singapore; ssummers@ntu.edu.sg (S.S.); RSCOTT@ntu.edu.sg (S.A.R.); 3 The ithree Institute, The University of Technology Sydney, Sydney, NSW 2007, Australia

**Keywords:** antibiotic susceptibility, WGS, phylogenetic analysis, DNA mismatch repair system

## Abstract

This study investigated genomic differences in Australian and Indian *Pseudomonas aeruginosa* isolates from keratitis (infection of the cornea). Overall, the Indian isolates were resistant to more antibiotics, with some of those isolates being multi-drug resistant. Acquired genes were related to resistance to fluoroquinolones, aminoglycosides, beta-lactams, macrolides, sulphonamides, and tetracycline and were more frequent in Indian (96%) than in Australian (35%) isolates (*p* = 0.02). Indian isolates had large numbers of gene variations (median 50,006, IQR = 26,967–50,600) compared to Australian isolates (median 26,317, IQR = 25,681–33,780). There were a larger number of mutations in the *mutL* and *uvrD* genes associated with the mismatch repair (MMR) system in Indian isolates, which may result in strains losing their efficacy for DNA repair. The number of gene variations were greater in isolates carrying MMR system genes or *exoU*. In the phylogenetic division, the number of core genes were similar in both groups, but Indian isolates had larger numbers of pan genes (median 6518, IQR = 6040–6935). Clones related to three different sequence types—ST308, ST316, and ST491—were found among Indian isolates. Only one clone, ST233, containing two strains was present in Australian isolates. The most striking differences between Australian and Indian isolates were carriage of *exoU* (that encodes a cytolytic phospholipase) in Indian isolates and *exoS* (that encodes for GTPase activator activity) in Australian isolates, large number of acquired resistance genes, greater changes to MMR genes, and a larger pan genome as well as increased overall genetic variation in the Indian isolates.

## 1. Introduction

*Pseudomonas aeruginosa* is a ubiquitous bacterium which can cause opportunistic or nosocomial infections in immuno-compromised patients [1]. *P. aeruginosa* commonly causes corneal (keratitis) [2], respiratory, burn and wound infections, and infections related to medical or surgical devices including ventilator-associated pneumonia [3,4]. *P. aeruginosa* corneal infections are usually related to contact lens wear, but other risk factors for keratitis in non-contact lens wearers include ocular trauma, ocular surgery, and prior ocular surface disease [5,6,7,8].

The prevalence of multi-drug resistant (MDR) or extensively drug resistant strains of *P. aeruginosa* reduces treatment options, significantly increasing morbidity rates [9]. *P. aeruginosa* is naturally resistant to some antibiotics due to the possession of specific resistance genes such as *catB* that confers chloramphenicol resistance and an inducible *ampC* which encodes for a β-lactamase that hydrolyses cephalothin and ampicillin, conferring resistance to β-lactams [10]. Additionally, the regulation of efflux pumps also contributes towards an elevated resistance to antibiotics [11]. For example, expression of the efflux pump MexAB-OprM contributes towards intrinsic resistance to a broad spectrum of antibiotics [12], whereas the efflux pump MexXY-OprM is involved in the adaptive resistance to aminoglycosides [13]. Other resistance mechanisms in *P. aeruginosa* include the acquisition of transferrable resistance determinants, including those associated with transposons and integrons [14]. Antibiotic resistance of *P. aeruginosa* varies according to the region where the strains have been isolated [15,16] presumably due to the prescribing practices, availability of antibiotics, and perhaps their use in animal husbandry. Various epidemiological studies have identified MDR *P. aeruginosa* from different infections and these isolates have acquired different resistance characteristics. For example, aminoglycoside resistance [17] and ciprofloxacin persistence [18] are found in cystic fibrosis isolates of *P. aeruginosa.* Some of these MDR strains are clonal and such clonal strains are often the predominant global clinical MDR isolates [19] which spread resistance characteristics into the wider population which enables clonal lineages to expand with time.

*ExoU* has been associated with virulence of *P. aeruginosa* at the ocular surface. *ExoU* is a phospholipase that causes mammalian cell death [20] and *exoU* possession is common in strains isolated from ocular infections [21]. There is a correlation between carriage of *exoU* and elevated resistance to fluoroquinolones and aminoglycosides [22]. *ExoU* is carried by strains on a genomic island that also contains resistance genes for a range of antibiotics [23].

In addition to the acquisition of resistance genes, bacteria can develop resistance through mutation of genes so that antibiotic targets are modified. Mutation rates are elevated in strains that carry mutations in DNA mismatch repair (MMR) systems and hence such mutator strains will normally carry more mutations than non-mutator strains [24]. In *P. aeruginosa*, the MMR system is composed of *mutS*, *mutL*, and *UvrD* genes [25]. Strains of *P. aeruginosa* isolated from the lungs of cystic fibrosis patients have alterations in the DNA MMR system and this has been correlated with multiple antimicrobial resistance [23].

In Australia, there is a tight regulation of prescribing antibiotics, and antibiotics can only be obtained legally with a prescription from a qualified healthcare professional according to the Therapeutic Goods Act 1989. In India, on the other hand, whilst branded antibiotics exist, other forms such as counterfeit, substandard, and ‘spurious’ antibiotics have been reported [26], making surveillance and regulation difficult [27]. While the antibiotic consumption per person in Australia and India in 2010 was approximately similar, there was a more rapid increase between 2000 and 2010 in India [28]. These differences may affect antibiotic resistance development.

The aim of the current study was to compare the phenotypic resistance and genetic characteristics associated with resistance between strains isolated from Australia and India to better understand the underlying factors that may lead to an increased resistance in *P. aeruginosa* strains associated with ocular infection.

## 2. Results

### 2.1. Antibiotic Susceptibility

The minimum inhibitory concentrations (MICs) and minimum bactericidal concentrations (MBCs) of the *P. aeruginosa* isolates were determined (Table 1). Strains showing intermediate resistance (I) as well as full resistance to antibiotics were categorized as resistant (R) for subsequent analyses. Based on the Centers for Disease Control and Prevention’s (CDC, Atlanta, GA, USA) definition of multi-drug resistance as “an isolate that is resistant to at least one antibiotic in three or more drug classes”, isolates 198, 202, 216, 217, 218, 219, 220, and 221 were deemed to be multi-drug resistant. Australian isolates 223, 224, 225, 227, 233, and 235 were also resistant to three antibiotics but these antibiotics were not of different classes. Isolates 176, 193, and 206 were sensitive to all antibiotics, but all other isolates were resistant to at least one antibiotic. Overall, Indian isolates were more resistant to antibiotics compared to Australian isolates. Among Australian isolates (*n* = 14), resistance was 78% for imipenem, 57% for ceftazidime, 50% for ciprofloxacin, 21% for piperacillin, 14% for levofloxacin, 7% for tobramycin, and no isolates were resistant to gentamicin or polymyxin. In contrast, resistance in Indian isolates (*n* = 12) was 75% for ciprofloxacin, 58% for imipenem, 50% for levofloxacin, tobramycin, and ceftazidime, 41% for piperacillin, 40% for gentamicin, and 25% for polymyxin.

### 2.2. General Features of the Genomes

The isolates after de novo assembly consisted of different numbers of contigs ranging from 50 for isolate 169 to 1917 for isolate 216. The average number of coding sequences was 6162 ± 359.2 for the Australian isolates and 6544 ± 889 for the Indian isolates. Isolates had an average of 66.1% G + C content. The tRNA copy number for the isolates ranged from 57 to 86 (which may vary between studies that use different assembly methods). The general features of the isolates are provided in Supplementary Table S1.

### 2.3. Acquired Resistance Genes

*P. aeruginosa* isolates were examined for horizontally acquired antibiotic resistance genes (Table 2) using the Resfinder database. Altogether, 33 different acquired antibiotic resistance genes for various classes of antibiotics including aminoglycosides, fluoroquinolones, beta-lactams were found in these isolates (Table 2).

An aminoglycoside resistance gene (*aph(3’)-IIb*), a beta-lactam resistance gene (*blaPAO*), a fosfomycin resistance gene (*fosA*), and a chloramphenicol resistance gene (*catB7*) were common to all isolates. The Australian isolates (123–182) had acquired only eight resistance genes, while the Indian isolates (188–221) had acquired 26 different resistance genes (Table 2). Five Indian isolates (198, 202, 217, 219, and 221, with large pan genomes) acquired the largest number of resistance genes. Of these five isolates, the pairs 198/219 and 202/221 had the most similar resistance gene profiles and each member of the pair were of the same sequence type, ST308 and ST316 respectively. As acquired resistance genes may be carried on integrons, the genomes of the *P. aeruginosa* isolates were analyzed for integrons using Integron Finder version 1.5.1. *qnrCV1* was associated with a class 1 integron in isolates 202 and 221 and a Tn3 transposon in isolates 198 and 219.

Several types of non-synonymous variations were found in the core genome of the keratitis *P. aeruginosa* isolates when compared with the reference genome of PAO1 (Table 3). These non-synonymous mutations included single nucleotide polymorphisms (SNPs), multi-nucleotide polymorphisms (MNPs), deletions, insertions, and complex variations (where more than one change occurred at one specific location compared to the reference strain). The total variations in the isolates ranged from 76,080 in isolate 206 to 22,536 in isolate 181. There was a median of 26,317 (IQR = 25,681–33,780) variations in the genomes of Australian isolates and a median of 50,006 (IQR = 26,967–50,600) in the Indian isolates (*p* = 0.09). Based on the grouping of core genome phylogeny, isolates within group 2 (198, 202, 219, 220, 221, 233) had the most variations. Isolate 206, which had a unique sequence type and was placed in a separate group by pan genome analysis, had an exceptionally high number of variations (76,080) and SNPs (67,271).

Non-synonymous mutations were assessed in resistance genes of the *P. aeruginosa* isolates (Supplementary Table S2). There were no large differences in the mutations in resistance genes of any of the isolates except the antibiotic efflux-related genes *opmH* and *rosC*. *opmH* had ≥9 mutations in all isolates except 127, 162,169, 202, 218, 220, and 221 (mostly isolates of group 2 of core and pan genome phylogenies except 127 and 218). *rosC* had 20 non-synonymous mutations including insertions/deletions in isolate 206, 11 in 233, and ≥5 mutations in isolates 162, 169, 176, 202, 216, 217, 219, 220, and 221 (mostly isolates of group 2 of core and pan genome phylogenies except 176, 216), but ≤3 mutations in isolates 123, 126, 123, 181, 182, 188, 189, 193, 198, and 218 (mostly isolates of group 1 of core and pan genome phylogenies except 198). Mutations in efflux genes encoding efflux pumps were also found, including *mexX*, *mexT*, *mexD*, *mexM*, and *mexY*, although there was no significant difference between two groups in the possession of mutations in these genes. All other mutations in the genes were random without any association to sequence type, phylogeny, or susceptibility to antibiotics.

### 2.4. Possession of exoU and Mutations in the DNA Mismatch Repair System

*ExoU* was present in the genomes of all isolates in group 2 (core and pan genome phylogenetic group) as well as isolates 123 and 127. All other isolates possessed *exoS* with the exception of isolate 126 which possessed both *exoU* and *exoS*. To address differences in the numbers of sequence variants between the isolates, the genes involved in the DNA mismatch repair (MMR) system *mutS* (that encodes a protein which binds to errors in DNA), *mutL* (that encodes a protein that works in synergy with MutS and activates UvrD), and *uvrD* (a DNA helicase active in DNA replication) were examined. The mutations in the MMR system included SNPs, indels, and complex variants. The number of mutations in *mutL* ranged from 1 to 2 and mutations in *mutS* (which ranged between 0 and 2) were found in seven isolates (Table 4). In *uvrD*, the number of mutations ranged between 0 and 5 (Table 4). *exoU* containing isolates possessed a median of two (IQR = 1–3) mutations in *mutL*, zero (IQR = 0–2) mutations in *mutS*, and four (IQR = 2–5) mutations in *uvrD*, whereas *exoS* containing isolates possessed a median of zero (IQR = 0–1) mutations in *mutL*, zero (IQR = 0–1) mutations in *mutS*, and two (IQR = 0–2) median mutations in *uvrD*. There were significant differences in the number of *mutL* (*p* = 0.0021) and *uvrD* (*p* = 0.02) mutations in *exoS* and *exoU* isolates but not with *mutS* (*p* = 0.3). Isolate 206, an *exoS* strain and an outlier in the core genome analysis, had one mutation in *mutL*. Details of mutations occurring in nucleotide and respective proteins are provided in Supplementary Table S3.

### 2.5. Sequence Type Analysis and Phylogenetics

All Australian isolates were of different sequence types (ST), except 225 and 227 which belonged to ST233. Among the 12 Indian isolates, one isolate was designated as belonging to a new sequence type, two isolates (198 and 219) belonged to ST308, two others (188 and 189) belonged to ST491, and three isolates (202, 220, and 221) belonged to ST316 (Table 5).

**Figure 1:**
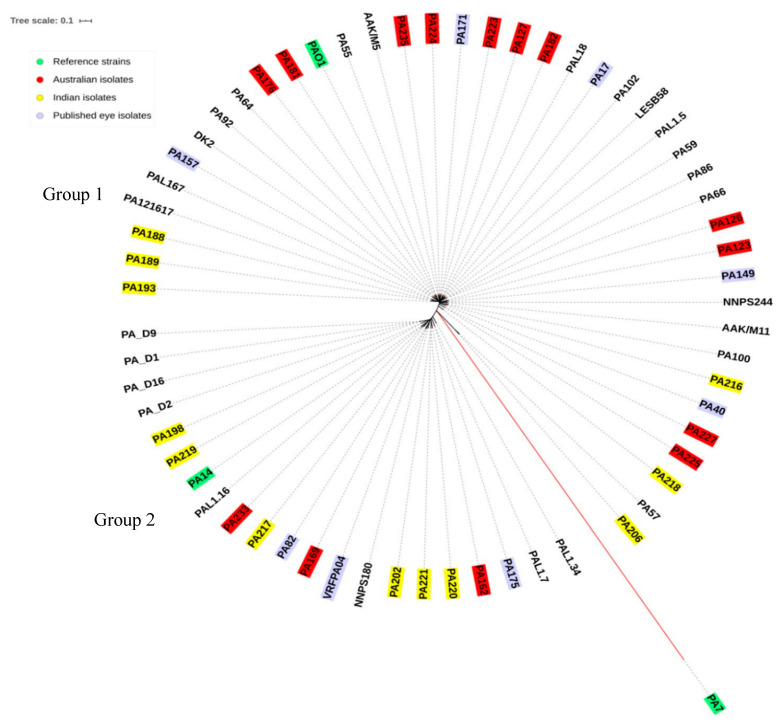
Core genome phylogeny of *P. aeruginosa* isolates using Parsnp. PAO1 was used as reference. PA7 and PA14 were also included.

The number of core and total or pan (or total) genes were reported from the statistical summary of Roary v3.11.2. The core genomes of the isolates were aligned using PA7 (Accession number NC_009656.1), PA14 (Accession number NC_004863.1), and PAO1 (Accession number NC_002516.1) as reference strains. The eight published genomes of *P. aeruginosa* isolates from eye as well as strains from other sources were also included. The core genes of published isolates are provided in Supplementary Table S4. The isolates were sub-grouped based on the number of core genes; isolates with a similar number of core genes were closely aligned and isolates with the same sequence type were grouped together. The core genomes formed two groups in the phylogenetic tree (Figure 1). Isolates in group 1 tended to have a larger number of core genes than isolates in group 2. Isolate 206, PA57, and PA7 were outliers based on core genome phylogeny. The Australian and Indian isolates had a similar number of core genes, whereas the Indian isolates had a larger number of pan genes (10,889) due to the acquisition of shell genes (genes present in two or more strains) (Table 4).

The phylogenetic relationships of these *P. aeruginosa* isolates were assessed by aligning their pan genome against PAO1 as a reference. The output generated using Roary showing the gene presence or absence in all isolates is provided in Supplementary Figure S1. This again divided the *P. aeruginosa* isolates into two major groups. Six multi-drug resistant Indian isolates (198, 202, 217, 219, 220, 221) and the VRFPA04 isolate (isolated from the cornea) were clustered in one group, which also contained the two Australian isolates 162 and 169. The Indian isolate 216 was categorized in a separate sub-group due to the large number of shell genes and possession of *exoS*.

**Figure 2:**
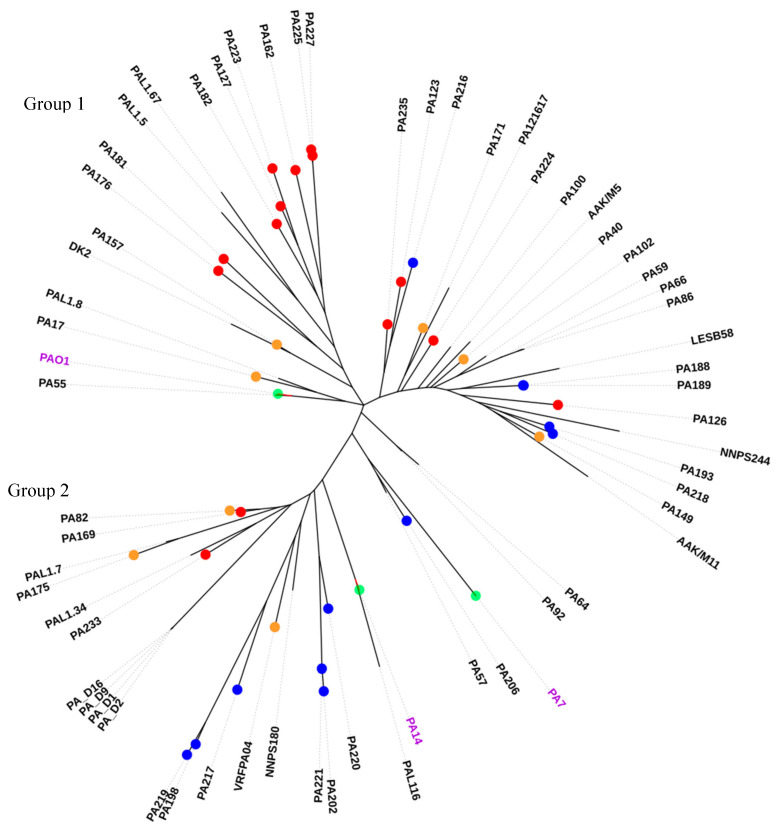
Pan genome phylogeny of *P. aeruginosa* isolates. Branches with no color representation indicate non-ocular isolates. Red color indicates Australian, blue color represents Indian, and orange color represents published eye isolates. Green color represents reference strains. Purple color represents reference strains.

The second group (group 2 of the pan genome analysis) included most of the Australian (123, 126, 127, 162, 176, 181, 182, 223, 224, 225, 227, 235) and Indian (188, 189, 193, 216 218) isolates along with reference strain PAO1 (Figure 2). Overall, the multi-drug resistant Indian isolates had a large pan genome (total of 10,889 genes obtained from the statistical summary in Roary v3.11.2). The pan genome grouping of isolates was broadly based on the number of pan (or total) genes and possession of either *exoU* or *exoS* in each group, except two Australian isolates 123 and 127 which were in group 2 but possessed *exoU*. The other exception to this grouping pattern was for isolates 181 and 182 which had large pan genomes and were clustered into group 1 but carried *exoS*.

The isolates of group 2 usually had a large number of pan genes and were *exoU+*. Isolates having similar numbers of pan genes were sub-grouped together. For example, isolate 193 (pan genes = 6084) and 218 (pan genes = 6001) were sub-grouped together. Isolate 218 had a similar number of pan genes to isolate 123 (pan genes = 6001), but isolate 218 possessed *exoS*, while 123 possessed *exoU*, and thus these were not grouped together. Isolates belonging to the same sequence type were also grouped together. The MDR isolates, the isolates with same STs, and isolates with large gene variations were clustered in one pan-group. The previously published isolates PA_D1, PA_D2, PA_D9, and PA_D16 with the same ST and those with large shell genes were grouped with the MDR isolates of the current study.

## 3. Discussion

This study investigated genomic differences in Australian and Indian *P. aeruginosa* isolates from keratitis. Phenotypically, more resistance was found in Indian isolates compared to Australian isolates as has been shown in previous studies [30,31]. Unregulated antibiotic use in India has been linked to increased antibiotic resistance [32]. Resistance to antibiotics is problematic even in the treatment of keratitis, where a topical application of antibiotics is used. Infection with antibiotic resistant strains results in prolonged infection [33], more severe keratitis [5], and an increase in the cost of treatment [34,35].

Indian *P. aeruginosa* strains harbored more resistance genes compared to Australian isolates, although *aph(3’)-IIb, blaPAO1 (fosA)*, and *catB7* were found in all isolates, which was consistent with previous studies [31,36]. *qnrVC1* was found in four Indian isolates but no Australian isolates. This fluoroquinolone resistance gene has not been previously reported in *P. aeruginosa* ocular isolates [31], but it has been reported in burns isolates and has been identified as being carried on an integron [37]. Similarly, in the current study *qnrVC1* was carried on a class 1 integron in isolates 202 and 221, but integrated into a Tn3 transposon in isolates 198 and 219. This gene has also been isolated from the high risk ST773 clone of *P. aeruginosa* from urine in Hungary [38]. High risk clones are isolates with high mutational rates in resistance genes and those that have acquired a large number of resistance genes. As previously described, resistance to fluoroquinolones in keratitis *P. aeruginosa* isolates was also due to mutations in the quinolone resistance determining regions of *gyrA* and *parC* [15]. Possession of *qnrVC1* and mutations in *gyrA* and *parC* were associated with high levels of fluroquinolone resistance. The possession of large numbers of acquired resistance genes by Indian isolates likely contributed to the higher rates of resistance of these isolates. The Indian isolates 198, 202, 217, 219, 220, and 221 also had a high number of gene variations which is an independent mechanism of resistance.

The aminoglycoside resistance gene *aph(6)-Id* which encodes for streptomycin resistance was found in six Indian isolates, including the four that carried *qnrVC1*, but in no Australian isolates. Previously, *aph(6)-Id* was found in only one Indian ocular isolate from 1997 [31], but has been found in cystic fibrosis *P. aeruginosa* isolates [39] and has been associated with the transposon Tn5393 on a plasmid in one strain of *P. aeruginosa* [39]. As streptomycin is no longer used in clinical treatment [40], this resistance may not be clinically relevant but does suggest environmental selection for the persistence of this gene. 

The total number of gene variants found in the Indian isolates 198, 202, 219, and 221 were greater than Australian isolates. However, there were a small number of SNPs found in the genes associated with resistance for these isolates. The Indian isolate 206 (NEWST) had a high number of SNPs in antibiotic resistance genes *mexC*, *mexD*, *mexM*, *mexX*, *mexS*, *opmE*, *mexP*, *mexK, oprJ*, *ampC*, *rosC*, and *mprF*. There was no difference in the mutations of other *mex* genes including *mexX*, *mexT*, *mexD*, *mexM*, and *mexY* between Australian and Indian isolates. Given that most isolates from both countries, whether they were sensitive or resistant, had a similar number of mutations in the resistance genes, it is likely that the resistance to antibiotics was related to the possession of acquired resistance genes rather than mutations in chromosomal genes.

In the Australian isolates, four out of the eight isolates (50%) carried *exoU*, while one isolate was both *exoU+/exoS+* and three (38%) were *exoS*+. In Indian isolates, 50% carried *exoU* and 50% carried *exoS*. A previous study has also shown an equal ratio of both genes [41] in keratitis isolates. The possession of the *exoU* genotype in *P. aeruginosa* ocular isolates has been related to elevated resistance to disinfectants [42], fluoroquinolones [43], and multiple antibiotics [41]. Furthermore, one study reported worst clinical outcomes and more resistance by *exoU* carrying isolates [43]. The isolates of this study showed similar findings because the *exoU+* isolates 198, 202, 217, 219, 220, 221, and 233 were also MDR.

The DNA mismatch repair system (MMR) in *P. aeruginosa* is based on the protein trimer MutS-MutL-UvrD and functions to correct errors and preserve the integrity of the genome [24,44,45]. The *mutH* component of MMR, which is important in other Gram negative bacteria, such as *E. coli* [46], has not been found previously in *P. aeruginosa* [25] and was not present in the isolates of the current study. Mutations in *mutS*, *mutL* and *uvrD* can reduce the ability of the bacterium to repair DNA lesions [46]. Strong mutator strains have defects in their MMR system and mutations in *mutS* predominate [47]. Mutations in the MMR can be a reason for the development of hypermutations in isolates. In cystic fibrosis, hypermutations were found to be a key factor in the development of MDR resistant *P. aeruginosa* strains [23]. Similar findings were found in this study where isolates 198, 202, 206, 219, and 221 had mutations in the MMR genes, and these isolates had an overall larger variation in their genomes. In the current study, isolates had more mutations in *mutL* and *uvrD,* suggesting the strains may not be strong mutators (which is usually associated with mutation in *mutS*), but nevertheless can undergo uncorrected genetic changes. Indeed, the *P. aeruginosa* isolates in the current study which had mutations in *mutL* and *uvrD* had greater numbers of SNPs, insertions and deletions, acquired genes, and had large pan genomes. Among these isolates, 198, 202, 219, and 221 possessed either the transposon Tn3 or class 1 integrons which carried the acquired genes. This also might be due to mutated MMR, as mutations in MMR genes increase the chances of horizontal gene transfer in mutator isolates [47]. The number of mutations in MMR was greater in *exoU* possessing isolates with large gene variations. *exoU* is carried on genomic islands [48,49] and these *exoU* carrying isolates had larger pan genomes with possession of mobile genetic elements. Therefore, the isolates with the mutated MMR systems may have a greater ability of strains to accumulate gene variations and the acquisition of *exoU*. Isolate 206, on the other hand, possessed *exoS* and was not MDR but possessed a large number of SNPs and a large pan genome with one mutation in each of *mutS* and *mutL*. Further in-depth studies are required to understand the influence of the MMR system on genomic changes in *P. aeruginosa*.

Analysis of the sequence types of the *P. aeruginosa* ocular isolates revealed the presence of three clones, two in the Indian and one in the Australian isolates. The isolates with the same STs had mostly the same phenotypic and genotypic features. The exception to this was isolate 220 that had acquired fewer resistance genes compared to the other two isolates 202 and 221 of ST316. Previously, five ocular *P. aeruginosa* strains from India isolated in 1997 were of sequence type ST308 [31]. The two isolates of ST308 in the current study, isolated in 2017 and 2018, had acquired more resistance genes compared to isolates from 1997 [31]. This indicates that the clonal isolates have continued to evolve over this time period, although the specific selection factors driving those changes are yet to be elucidated. None of the isolates were collected from the same patient. The majority of the isolates with the same STs grouped in the same phylogeny including previously published isolates (PA_D1, PA_D2, PA_D9, P_D16) with ST1971.

Core and pan genome phylogenies of the isolates produced two almost identical groups, which was in agreement with previously published studies [31,50]. Both phylogenies included isolates from either Australia or India, but those in group 2 tended to be the MDR Indian isolates and possessed higher numbers of antibiotic resistance genes. About 65% of all ocular isolates grouped together which indicated less diversity in the ocular *P. aeruginosa* isolates [31,51,52]. The grouping of MDR strains from this study with PA14 along a MDR ocular isolate VRFPA04 [36] in both core genome and pan genome analysis, and the grouping of the sensitive strains with PAO1 along the commonly studied cystic fibrosis isolates DK2 and LESB58, was similar to a previous study examining older isolates from India and Australia [31]. Isolate 206, which had the smallest number of core genes and was of a new sequence type, was an outlier in the core genome phylogeny similar to the taxonomic outlier PA7 [53]. However, isolate 206 was grouped together with other isolates in the pan genome because it had acquired a large number of genes. Acquired genes are part of the pan rather than the core genome [53] and the presence of larger pan genomes in MDR *P. aeruginosa* isolates points towards the acquisition of new genes [54]. Previously, a smaller core genome size of 4910 genes has been reported in ocular *P. aeruginosa* isolates [31]. However, the current study found a core genome size similar to *P. aeruginosa* from different sources, comprising 5316–5233 genes [55,56]. The core genome (which is almost 90% of total genome) refers to the conserved genes present in a species [57] which might differ in each individual strain within that species. Additionally, SNPs can be a result of poor sequencing quality and hence it is important to have a good sequencing depth at those positions to identify them as a mutation rather than sequencing error [58]. Grouping of all the isolates including ocular and non-ocular remained the same in both core and pan genome phylogeny.

## 4. Materials and Methods

### 4.1. P. aeruginosa Strains and Susceptibility Testing

Twenty-six *P. aeruginosa* keratitis isolates, eight isolated in Australia from 2004 to 2006, six from 2018 and 2019 (total 14 Australian isolates), and twelve isolated in India between 2017 and 2018, were included in this study. These isolates were selected from a larger collection of strains based on their antibiotic susceptibilities (those phenotypically resistant to multiple antibiotics, some resistant to one or multiple antibiotics, and some which were sensitive to all antibiotics). The susceptibilities of Australian strains (2004–2006) included in this study have been previously published [29]. Strains were selected after comparing their susceptibilities to antibiotics that are used to treat ocular infections. For genetic comparisons, the data of 34 *P. aeruginosa* isolates from eyes and other sources were also included. The general characteristics of these isolates are described in Supplementary Table S4. The genomes of these isolates were downloaded from the NCBI database and reannotated for this study using the same parameters as of the isolates of this study to avoid any bias in results.

The minimum inhibitory concentration (MIC) and minimum bactericidal concentration (MBC) of various antibiotics which are commonly used to treat *P. aeruginosa* keratitis [16] were assessed for the isolates using the broth microdilution method in 96-well plates following the Clinical and Laboratory Standard Institute guidelines [59]. The antibiotics tested were ciprofloxacin, levofloxacin, gentamicin, ceftazidime (Sigma-Aldrich, St. Louis, MO, USA), polymyxin B (Sigma-Aldrich, Vandtårnsvej, Søborg, Denmark), tobramycin, piperacillin (Cayman Chemical Company, Ann Arbor, MI, USA), and imipenem (LKT Laboratories Inc., St. Paul, MN, USA). The susceptibility results were interpreted using the EUCAST v9 [60] and CLSI [61] 2017 breakpoints.

### 4.2. Genomic Sequencing

DNeasy Blood and Tissue Kits (Qiagen, Hilden, Germany) were used for DNA extraction as per the manufacturer’s recommendations. The Nextera XT DNA library preparation kit (Illumina, San Diego, CA, USA) was used to prepare paired-end libraries. All the libraries were multiplexed on one MiSeq run. FastQC version 0.117 (https://www.bioinformatics.babraham.ac.uk/projects/fastqc) was used to assess the quality of sequenced genomes using raw reads. Version 0.38 of Trimmomatic [61] was used for trimming the adapters from the reads following de novo assembly using Spades v3.13.0 [62]. Genomes were annotated using Prokka v1.12 [63].

Sequence types were investigated using PubMLST https://pubmlst.org/. Pan genomes of the *P. aeruginosa* isolates were analyzed using Roary v3.11.2 [64] using PAO1 as a reference, while core genome phylogeny was constructed using Harvest Suite Parsnp v1.2 [65] with strains PAO1, PA7, and PA14 used as reference strains. The output file ‘genes_ presence_absence’ was used to compare the *P. aeruginosa* isolates. Acquired resistance genes were identified using the online database Resfinder v3.1 (Centre for Genomic Epidemiology, DTU, Denmark) [66]. Integron Finder v1.5.1 was used to identify any integrons present in the isolates. Mutations in the genes were detected using Snippy V2 [67]. Isolates with same sequence types were compared for nucleotide similarities using the MUMmer online web tool (http://jspecies.ribohost.com/jspeciesws/#analyse).

Using the Pseudomonas genome database (http://www.pseudomonas.com) and comprehensive antibiotic resistance database (https://card.mcmaster.ca), 76 genes related to *P. aeruginosa* resistance were selected to investigate the presence of single nucleotide polymorphisms. All isolates were analyzed for the presence of the type III secretion system associated virulence factors *exoU* and *exoS* using the BlastN database.

### 4.3. Statistical Analysis

The statistical analysis was performed using GraphPad Prism v8. Medians were calculated with the ‘descriptive statistics’ option during analysis of variance (ANOVA). *P*-values less than 0.05 were considered as significant. Fischer’s Exact test was used to find the difference between acquired genes. To analyze the significant difference in the DNA mismatch repair genes between *exoU* and *exoS* isolates and gene variations in the isolates, the Mann–Whitney test was used.

## 5. Conclusions

Indian isolates and Australian isolates were clearly distinct in carrying a type III secretion system related to *exoU* and *exoS*. There was an association in the isolates for carrying acquired resistance genes with a large number of pan genes. Indian isolates were more resistant to antibiotics compared to Australian isolates. Additionally, isolates of *P. aeruginosa* from ocular infection had a large number of genetic variations (mutations) and a mutated mismatch repair system. However, the isolates collected from the same region or time will give a clearer idea of these differences.

## Figures and Tables

**Table 1:** Minimum inhibitory concentration (MIC) and minimum bactericidal concentration (MBC) of antibiotics to *Pseudomonas aeruginosa* keratitis isolates.

*Strain Number*	Fluoroquinolones *		Aminoglycosides		β-Lactams			Poly-Peptide
	2nd Generation	3rd Generation			Penicillin 4th Generation	Carba-Penem	Cephalosporin 3rd Generation	
	Cipro µg/mL ≤1, 2, ≥4 ^#^	Levo µg/mL ≤2, 4, ≥8	Genta µg/mL ≤4, 8, ≥16	Tobra µg/mL ≤4, 8, ≥16	Pipera µg/mL ≤16	Imi µg/mL ≤2, 4, ≥8	Ceftaz µg/mL ≤8, 16, ≥32	PMB µg/mL ≤2, 4, ≥8
	MIC/ MBC	MIC/ MBC	MIC/ MBC	MIC/ MBC	MIC/ MBC	MIC/ MBC	MIC/ MBC	MIC/ MBC
123	1/1	1/1	0.25/0.5	4/4	8/16	4(I)/8	2/2	1280(R)/1280
126	0.5/1	0.5/1	0.5/1	0.25/0.5	8/16	8(R)/16	128(R)/256	1/1
127	1/2	0.25/1	2/4	32(R)/128	4/16	4(I)/8	128(R)/256	0.5/1
162	0.5/1	0.5/1	0.25/0.5	0.25/1	8/8	4(I)/4	2/4	0.25/0.5
169	2(I)/4	0.25/0.5	0.25/0.5	0.25/0.5	4/8	2/4	1/2	0.25/0.25
176	0.5/1	0.25/0.5	0.25/0.5	0.25/0.5	4/8	2/8	2/4	0.25/0.5
181	1/4	0.25/0.5	0.25/0.5	0.25/0.5	32(R)/64	4(I)/8	16(I)/32	0.5/1
182	1/2	0.25/0.5	0.25/0.5	0.25/0.5	4/8	8(R)/16	1/2	0.25/0.5
223	64(R)/128	1/2	0.5/1	0.5/1	160(R)/320	1/2	16(I)/32	2/4
224	16(R)/32	1/2	0.25/0.5	0.25/0.5	8/16	64(R)/128	16(I)/32	1/2
225	64(R)/128	16(R)/32	0.5/2	1/2	16/32	64(R)/128	8/16	0.25/0.5
227	64(R)/128	64(R)/128	0.5/1	0.25/1	16/32	16(R)/32	16(I)/32	0.25/0.5
233	8(R)/16	1/2	1/2	105/1	16/32	4(I)/8	160(I)/320	0.5/1
235	16(R)/32	0.5/1	2/4	0.5/1	64(R)/128	4(I)/8	64(I)/128	0.25
188	2(I)/4	1/2	0.5/1	32(R)/64	16/65	0.5/1	4/8	2/4
189	0.25/1	1/2	0.25/0.5	16(R)/32	4/8	2/1	8/16	2/4
193	1/1	0.25/1	0.25/0.25	0.25/0.5	4/8	2/4	2/2	0.5/1
198	1280(R)/2560	320(R)/1280	2560(R)/5120	16(R)/16	8/8	1/2	8/8	4(I)/4
202	640(R)/1280	320(R)/640	8(I)/32	320(R)/640	16/64	8(R)/32	8/32	0.25/0.25
206	1/1	0.5/0.5	1/1	0.25/0.5	8/8	2/4	2/4	0.25/0.5
216	64(R)/128	4 (I)/8	1/2	0.5/2	160(R)/320	16(R)/32	64(R)/128	64(R)/128
217	64(R)/128	32(R)/64	1/2	1/2	64(R)/128	8(R)/16	32(R)/64	0.25/1
218	8(R)/16	1/2	0.5/1	0.5/1	160(R)/320	8(R)/16	64(R)/128	1/4
219	≥5120(R)/≥5120	640(R)/1280	≥5120(R)/≥5120	1280(R)/2560	2560(R)/5120	40(R)/80	16(I)/32	0.25/1
220	2(I)/4	0.25/0.5	0.5/1	0.5/1	0.25/0.5	8(R)/16	160(R)/320	8(R)/16
221	2560(R)/5120	2560(R)/5120	2560(R)/5120	2560(R)/5120	64(R)/128	16(R)/32	32(R)/64	0.25/1

Data for Australian isolates (shaded in gray). Data for 123–182 is from a previously published study [29]. Strains 188–221 were Indian keratitis isolates. R = resistant, I = intermediate resistance. * Cipro = Ciprofloxacin, Levo = Levofloxacin, Genta = Gentamicin, Tobra = Tobramycin, Pipera = Piperacillin, Imi = Imipenem, Ceftaz = Ceftazidime, PMB = Polymyxin B; ^#^ = Antibiotic breakpoints for sensitive, intermediate, resistant classifications.

**Table 2:** Acquired resistance genes in *P. aeruginosa* isolates from India and Australia.

Genes	Australian Isolates														Indian Isolates											
	123	126	127	162	169	176	181	182	223	224	225	227	233	235	188	189	193	198	202	206	216	217	218	219	220	221
Aminoglycoside resistance genes																										
*aph(3’)-IIb*																										
*aph(6)-Id*																								l;		
*rmtD2*																										
*rmtB*																										
*aph(3’)-VI*																										
*aph(3’)-lIb*																										
*aph(3’’)-Ib*																										
*aac(6’)-Ib3*																										
*aac(3)-IId*																										
*aadA1*																										
*aac(6’)-Ib-cr*																										
Fluoroquinolone resistance genes																										
*crpP*																										
*qnrVC1*																										
Beta-lactamase resistance genes																										
*blaPAO*																										
*blaLCR-1*																										
*blaOXA-485*																										
*blaOXA-486*																										
*blaOXA-488*																										
*blaOXA-396*																										
*blaOXA-395*																										
*blaOXA-50*																										
*blaOXA-10*																										
*blaTEM-1B*																										
*blaVIM-2*																										
*blaPME-1*																										
*blaPAU-1*																										
Sulphonamide, tetracycline, macrolide, fosfomycin, and chloramphenicol resistance genes																										
*sul1*																										
*tet(G)*																										
*mph(E)*																										
*mph(A)*																										
msr(E)*																										
*fosA*																										
*catB*																										

* *msr(E) encodes macrolide and lincosamide resistance*. Isolates shaded in grey indicate Australian strains. *Black color represents gene presence*.

**Table 3:** Frequency of different types of variation in the genes of *P. aeruginosa* isolates.

*P. aeruginosa* Isolates	Total Variants	Variant Complex	Variants Insertions	Variants Deletions	Variants MNP	Variant SNP
123	28,279	1593	187	163	398	25,938
126	26,258	1416	164	159	355	24,164
127	25,760	1362	163	176	391	23,668
162	50,999	3481	281	257	951	46,029
169	50,283	3359	269	245	922	45,488
176	26,065	1372	168	161	342	24,022
181	22,536	1063	162	133	283	20,895
182	25,684	1359	172	180	368	23,605
223	25,672	1358	167	176	402	23,568
224	26,376	1435	163	165	353	24,260
225	28,070	1566	167	156	385	25,796
227	28,000	1560	162	154	370	25,754
233	52,392	3590	285	263	956	47,298
235	24,919	1349	162	171	354	22,883
188	25,833	1435	164	154	351	23,729
189	25,910	1458	165	155	365	23,767
193	26,567	1445	180	147	389	24,406
198	50,631	3503	280	236	945	45,667
202	49,981	3461	257	236	902	45,125
206	76,180	6449	336	371	1653	67,271
216	28,166	1548	183	164	433	25,838
217	51,119	3575	290	226	944	46,084
218	29,161	1676	182	181	430	26,692
219	50,507	3484	273	237	925	45,588
220	50,180	3452	267	234	894	45,332
221	50,030	3477	260	237	906	45,150

SNP = single nucleotide polymorphism; MNP = multi-nucleotide polymorphism. Isolate numbers highlighted in gray are from Australia.

**Table 4:** Possession of *exoU* and *exoS* and number and type of non-synonymous mutations in the mismatch repair system genes in *P. aeruginosa* isolates.

*P. aeruginosa* Isolates	Type III Secretion System Genes	*mutL*	*mutS*	*uvrD*
123	*exoU*	1 SNP	0	1 complex
126	*exoU/exoS*	0	0	0
127	*exoU*	0	1 MNP	1 MNP, 1 complex
162	*exoU*	1 SNP	0	2 SNP, 1 MNP, 2 complexes
169	*exoU*	1 SNP	1 complex	2 SNP, 2 MNP, 1 complex
176	*exoS*	1 SNP	0	1 SNP
181	exoS	0	0	0
182	*exoS*	0	0	1 MNP 1complex
223	*exoS*	0	1 SNP	1 MNP, 1 complex
224	*exoS*	1 SNP	0	1 MNP, 1 complex
225	*exoS*	0	0	2 SNP, 2 MNP, 1 complex
227	*exoS*	0	0	2 SNP, 2 MNP, 1 complex
233	*exoU*	0	0	1 MNP, 1 complex
235	*exoS*	0	0	0
188	*exoS*	0	0	1 MNP, 1 complex
189	*exoS*	1 SNP	0	1 MNP, 1 complex
193	*exoS*	0	0	0
198	*exoU*	2 SNP	0	1 SNP, 3 complexes
202	*exoU*	1 SNP	1 complex	1 SNP, 2 MNP, 2 complexes
206	*exoS*	1 MNP	1 complex	0
216	*exoS*	0	0	0
217	*exoU*	2 SNP	1 complex	1 SNP, 2 MNP, 1 complex
218	*exoS*	0	0	0
219	*exoU*	2 SNP	0	1 SNP, 1 MNP, 2 complexes
220	*exoU*	1 SNP	1 complex	1 SNP, 2 MNP, 2 complexes
221	*exoU*	1 SNP	1 complex	1 SNP, 2 MNP, 2 complexes

SNP = single nucleotide polymorphism, MNP = multinucelotide polymorphism. Isolates shaded in grey indicate Australian strains.

**Table 5:** Sequence types of *P. aeruginosa* isolates.

*P. aeruginosa* Isolates	Sequence Types	Core Genes	Shell Genes	Pan/Total Genes
123	ST218	5496	508	6004
126	ST2726	5483	712	6195
127	ST845	5483	938	6421
162	ST298	5439	905	6344
169	ST1027	5456	694	6150
176	ST709	5547	1112	6659
181	ST244	5588	1047	6662
182	ST27	5486	1096	6582
223	ST17	5471	1232	6703
224	ST168	5483	607	6090
225 ^¤^	ST233	5515	1338	6853
227 ^¤^	ST233	5493	1304	6797
233	NEWST	5440	624	6064
235	ST262	5470	540	6010
188 *	ST491	5490	535	6025
189 *	ST491	5492	531	6023
193	ST760	5490	594	6084
198 †	ST308	5454	1428	6882
202 #	ST316	5425	1505	6930
206	NEWST	5331	1084	6415
216	ST1527	5480	1488	6968
217	ST1047	5448	1173	6621
218	ST3083	5513	488	6001
219 †	ST308	5451	1796	7247
220 #	ST316	5430	948	6378
221 #	ST316	5425	1511	6936
PA7	ST1196	3599	4586	8185
PA14	ST253	5436	790	6226

Gray shading denotes Australian isolates. *, **†**, **#**, ^¤^ indicates strains belong to the same sequence types (STs).

## Supplementary Materials

The following are available online at https://www.mdpi.com/2079-6382/9/9/600/s1, Table S1: Details of the *Pseudomonas aeruginosa* isolates used in the current study. Table S2: Gene variations of resistance genes in *Pseudomonas aeruginosa* isolates (the gray-shaded strains were isolated from Australia). Nucleotide accession: The nucleotide sequences are available in the GenBank under the Bio project accession number PRJNA590804. Table S3: Types of mutations in the mismatch repair system; Table S4: Genomics features of *P. aeruginosa* isolates; Figure S1: Pan-genome phylogenetic tree. The data on the right of the figure shows the presence and absence of genes. The tree was built using the genome of PAO1 as a reference.
