# Supplementary material for: Antibiotic Resistance Characteristics of Pseudomonas aeruginosa Isolated from Keratitis in Australia and India
Source: Antibiotics (Basel). 2020 Sep 14;9(9):600. doi: 10.3390/antibiotics9090600 (PMC7559795; doi:10.3390/antibiotics9090600)
Supplement: Supplementary file 1 [file antibiotics-09-00600-s001.pdf]

S 1. Details of the *Pseudomonas aeruginosa* isolates used in the current study.

***P. aeruginosa* Isolates**	**Source**	**Region**	**Year of isolation**	**% GC**	**No. of contigs**	**Total sequence length (bp)**	**CDSs (total)**	**tRNAs**
123	Cornea	Australia	2005	66.4	86	6,357,971	5858	57
126	Cornea	Australia	2005	66.4	81	6,470,807	6007	57
127	Cornea	Australia	2005	66.4	86	6,357,971	5858	57
162	Cornea	Australia	2006	66.2	87	6,639,257	6131	59
169	Cornea	Australia	2006	66.4	50	6,397,665	5918	57
176	Contact lens	Australia	2004	65.8	305	7,065,610	6672	59
181	Cornea	Australia	2006	65.7	122	7,109,511	6619	63
182	Cornea	Australia	2004	66.1	58	6,836,159	6392	63
223	Cornea	Australia	2018	66.6	108	6,923,150	6408	70
224	Cornea	Australia	2018	66.4	151	6,418,520	5811	67
225	Cornea	Australia	2018	65.8	294	7,287,087	6607	68
227	Cornea	Australia	2018	65.6	102	7,141,983	6529	68
233	Cornea	Australia	2019	66.3	92	6,349,374	5745	65
235	Cornea	Australia	2019	66.1	56	6,279,891	5719	68
188	Cornea	India	2017	66.5	56	6,329,075	5818	58
189	Cornea	India	2017	66.5	59	6,329,723	5820	58
193	Cornea	India	2017	66.3	66	6,369,452	5888	61
198	Cornea	India	2017	66	119	7,101,775	6727	59
202	Cornea	India	2017	66	368	7,192,476	6883	61
206	Cornea	India	2017	66.4	55	6,535,880	6047	58
216	Cornea	India	2018	66.1	1917	8,328,371	8943	86
217	Cornea	India	2018	66.1	132	6,886,566	6482	58
218	Cornea	India	2018	66.4	77	6,375,153	5840	58
219	Cornea	India	2018	65.9	166	7,456,853	7112	59
220	Cornea	India	2018	66.3	90	6,653,669	6144	57
221	Cornea	India	2018	65.6	294	7,205,091	6829	59

Supplementary Table 2. Gene variations of resistance genes in *Pseudomonas aeruginosa* isolates (the grey shaded strains were isolated from Australia).

Gene name	Mechanism	*Pseudomonas aeruginosa*																									
		**Number of Single Nucleotide polymorphism**																									
		123	126	127	162	169	176	181	182	223	224	225	227	233	235	188	189	193	198	202	206	216	217	218	219	220	221
triA	**Antibiotic efflux**	1	1	1	2	2	1	0	0	1	0	0	0	3	0	0	0	3	5	3	1	1	4	0	5	3	3
triB		0	0	1	0	0	1	1	0	1	0	0	0	1	1	1	1	1	0	0	1	2	0	0	0	0	0
triC		0	0	0	1	1	2	0	0	0	0	0	0	2	0	0	0	0	2	2	0	0	2	0	2	2	3
mexR		0	0	0	1	1	0	0	2	0	1	0	0	1	0	0	0	1	1	1	2	0	1	1	1	1	1
mexA		0	1	0	0	0	0	0	0	0	0	0	0	0	0	0	0	1	0	0	0	0	0	0	0	0	0
mexB		0	0	0	0	0	0	0	0	0	0	0	0	0	2	0	0	1	2	1	1	0	1	0	2	1	1
oprM		0	0	0	0	0	0	0	0	0	0	0	0	0	0	0	0	0	0	0	0	0	0	0	0	0	0
farB		1	0	0	0	0	1	0	0	0	0	1	1	0	2	1	1	0	1	1	0	0	0	0	1	1	1
lrfA		3	4	4	6	6	5	6	7	3	3	5	4	5	6	3	3	3	8	5	8	3	7	5	8	5	5
lrfA		3	2	3	3	3	3	2	2	3	4	3	3	1	3	2	0	2	2	3	4	4	2	3	2	2	3
mexM		4	4	6	3	3	3	2	5	6	6	6	6	3	3	6	6	5	4	3	8	4	4	4	4	3	3
mdtC		3	3	3	1	2	1	0	2	3	2	3	3	1	3	3	3	4	2	1	3	2	3	0	2	1	1
cysB		0	0	0	0	0	0	0	0	0	0	0	1	0	1	0	0	0	0	0	0	0	0	0	0	0	0
mexY		2	1	1	5	2	2	2	1	1	3	1	1	4	2	3	3	3	5	2	4	1	2	1	5	2	2
mexX		3	3	3	4	3	3	3	3	3	3	2	2	4	3	2	2	3	4	4	7	3	4	3	4	4	4
macA		1	1	1	1	1	1	1	1	1	0	1	1	1	1	0	0	1	1	1	1	1	1	1	1	1	1
macB		1	2	1	3	1	2	1	1	1	1	1	1	2	1	2	2	2	1	2	2	2	0	2	1	2	2
opmQ		3	3	1	4	4	1	2	1	1	1	3	3	3	4	1	1	2	5	2	4	2	4	3	5	2	2
mexS		1	1	1	1	1	1	2	1	1	1	1	1	1	1	1	1	1	2	1	7	2	1	1	2	1	1
mexE		0	0	0	1	0	0	0	0	0	0	0	0	0	0	0	0	0	0	1	0	0	1	0	0	1	1
mexF		0	0	0	0	1	0	0	1	0	0	0	0	0	0	0	0	0	0	1	1	0	0	0	0	1	1
mexT		2	2	2	2	1	3	3	2	3	2	1	3	2	2	2	2	2	1	2	3	3	2	1	1	2	2

oprN		0	0	0	1	1	0	0	0	0	0	0	0	1	1	1	1	0	1	1	5	2	1	0	1	1	1
adeC		0	6	0	5	4	1	5	0	0	7	0	0	4	0	0	0	5	0	0	6	0	1	4	0	0	0
muxC		0	1	0	1	0	0	2	2	0	1	1	1	0	1	1	1	0	0	0	1	0	0	0	0	0	0
muxB		0	0	0	0	0	0	0	0	0	0	0	0	1	1	1	1	1	0	0	0	0	0	0	0	0	0
muxA		1	2	1	1	1	0	1	1	1	1	3	3	1	1	2	2	1	1	1	1	1	1	1	1	1	1
opmA		2	1	1	6	5	0	5	1	1	0	1	1	3	0	0	0	3	3	3	5	3	4	1	3	5	5
taeA		1	3	1	2	3	1	1	1	1	2	2	2	4	1	2	2	2	1	3	4	2	3	2	1	3	3
farB		1	0	0	1	0	0	0	1	0	0	2	2	1	1	1	1	2	0	0	2	1	0	3	0	0	0
opmE		3	3	4	0	4	5	0	4	4	3	3	3	3	6	4	4	5	3	3	9	5	5	4	3	3	3
mexQ		4	5	2	2	2	1	0	2	2	2	1	1	2	4	1	1	2	4	2	3	4	5	0	4	2	2
mexP		0	2	1	1	2	2	0	2	1	2	1	1	1	0	1	1	0	2	2	5	2	1	0	2	2	2
nalD		0	0	0	0	0	0	0	0	0	0	0	0	0	0	0	0	0	0	1	0	0	0	0	0	1	1
mexK		0	0	1	2	4	0	0	2	1	0	1	1	1	2	1	1	0	1	1	8	1	1	1	1	1	1
mexZ		0	0	0	0	0	0	0	1	0	0	0	0	0	0	0	0	0	1	1	2	1	0	0	1	0	1
mexJ		2	0	0	2	2	1	0	1	0	0	0	0	3	1	0	0	0	2	2	3	0	3	2	2	2	2
mexL		1	1	0	1	1	0	0	1	0	0	0	0	1	0	0	0	0	1	1	1	0	1	1	1	1	1
adeC		1	2	2	2	3	0	2	1	0	1	2	2	2	2	0	0	0	1	2	2	0	0	0	0	2	2
mexG		0	0	0	0	1	0	0	0	0	0	0	0	0	0	0	0	0	1	0	3	0	0	0	1	0	0
mexH		0	0	0	2	0	0	1	0	0	0	0	0	2	1	0	0	0	1	1	2	0	1	2	1	1	1
mexI		1	0	0	1	1	0	2	0	0	2	0	0	1	0	0	0	0	2	1	0	0	0	1	1	1	1
opmD		1	1	1	2	2	0	1	0	1	0	0	0	3	0	0	0	2	2	2	4	1	3	3	2	2	2
mexV		3	2	1	3	3	1	1	2	1	2	2	2	2	1	2	2	2	2	2	4	1	3	1	2	2	2
mexW		0	4	1	1	3	1	1	0	2	1	1	1	3	2	2	2	1	2	2	2	0	3	2	2	2	2
Yjjk		2	2	2	2	2	2	2	2	2	2	2	2	1	2	2	2	2	0	1	3	2	2	2	0	1	1
oprJ		1	1	1	2	0	2	1	1	1	0	1	1	0	1	1	1	0	0	0	8	1	1	0	0	0	0
mexD		1	3	2	2	2	3	1	1	2	1	1	2	4	2	2	2	2	2	2	11	3	4	2	2	2	2
mexC		2	1	0	4	6	1	4	0	0	1	2	2	5	1	1	1	3	7	5	10	3	4	2	7	5	5
nfxB		0	0	0	0	0	0	0	0	0	0	0	0	0	1	0	0	0	0	0	1	0	0	0	0	0	0
opmH		10	10	1	0	0	10	10	11	1	0	11	11	12	0	10	10	10	10	1	9	10	11	1	10	1	1

emrE		0	0	0	0	0	0	0	0	0	0	0	0	0	0	0	0	0	1	0	1	0	0	0	1	0	0
msbA		4	4	0	4	4	0	2	0	0	1	2	2	4	1	0	0	0	4	5	3	1	4	0	4	5	5
adeC		2	4	2	3	3	0	1	0	3	4	1	1	2	1	4	4	0	3	4	4	0	5	1	3	4	4
farB		3	3	4	5	4	3	3	3	4	3	3	3	4	3	4	4	0	3	3	4	3	3	3	3	3	3
rosB		1	0	2	1	1	1	1	0	2	1	1	1	3	1	3	3	1	2	2	1	0	1	2	2	2	2
catB7	**Antibiotic inactivation**	6	4	0	4	3	2	0	2	0	2	3	3	4	4	2	2	3	4	4	2	3	3	4	4	4	4
fosA		0	0	1	0	0	1	0	0	1	0	1	1	0	1	1	1	0	0	0	0	0	1	0	0	0	0
ampR		2	0	0	3	0	0	0	0	0	0	0	1	2	0	0	0	1	2	3	2	0	2	0	2	3	3
ampC		1	1	2	4	6	3	0	3	2	1	1	0	4	2	0	0	2	5	4	12	2	4	4	5	4	4
Aph(3′)-IIb		0	0	0	0	1	1	1	1	0	1	1	1	1	1	1	1	1	2	1	4	0	2	1	2	1	1
OXA-50		3	1	0	3	2	1	4	2	0	1	2	1	2	1	1	1	2	2	2	4	0	2	0	2	2	2
gyrB	**Antibiotic target alternation**	0	0	0	0	0	0	0	0	0	0	0	0	1	1	0	0	0	0	0	0	0	0	0	0	1	0
alaS		2	0	0	1	0	0	0	0	0	1	0	0	2	2	0	0	0	1	0	1	2	0	2	1	0	0
pmrC		3	3	3	3	3	3	3	2	3	2	3	3	3	3	1	1	2	3	4	3	4	3	2	3	4	4
mfd		2	2	0	3	1	0	1	0	0	0	2	2	1	1	0	0	0	2	1	3	0	1	1	2	1	1
gyrA		0	0	1	1	0	0	0	0	1	2	1	1	1	0	0	0	0	1	1	0	0	1	0	1	1	1
rocS		1	1	2	8	10	7	2	1	2	1	2	2	11	1	0	0	2	8	8	20	8	5	3	8	8	8
tufA		0	0	0	0	0	0	0	0	0	0	0	0	0	0	0	0	0	0	0	0	0	0	2	0	0	0
tufB		0	1	0	0	0	0	0	0	0	0	0	0	0	0	0	0	0	0	0	0	0	0	0	0	0	0
ileS		1	1	1	2	2	1	1	0	1	4	1	0	3	2	3	3	2	3	2	2	2	2	2	2	2	2
parC		1	0	0	1	1	0	0	0	0	0	0	0	1	0	0	0	0	2	2	1	1	1	0	2	1	2
parE		0	0	1	1	1	0	0	0	1	0	0	0	1	3	0	0	0	1	1	1	2	1	0	1	2	1
pmrF		0	0	0	1	0	0	0	0	1	0	0	0	0	0	0	0	0	0	0	3	0	0	0	0	0	0
arnA		2	3	1	2	3	1	2	1	1	0	3	3	4	0	2	2	1	3	3	6	1	3	3	3	3	3
mprF		0	4	1	2	8	1	1	1	1	1	3	3	5	1	3	3	5	6	2	9	6	6	2	6	2	2

Supplementary Table S3. Types of mutations in the mismatch repair system.

P. aeruginosa **isolates**	**Mutation in MMR genes**		
	mutL	***mutS***	***uvrD***
123	1SNP(missense_variant c.1034C>T p.Pro345Leu)	1SNP(missense_variant c.232G>A p.Val78Ile)	1 complex(missense_variant c.705_709delGCGGAinsACGGG p.Ile237Val)
126	0	0	0
127	0	1 MNP (missense_variant c.559G>T p.Ala187Ser)	1 MNP (missense_variant c.1985_1986delGTinsAC p.Ser662Asn) (1 Complex (missense_variant c.1985_1986delGTinsAC p.Ser662Asn)
162	1 SNP (missense_variant c.1784A>G p.Asn595Ser)	0	2 SNP (c.1031A>G p.Lys344Arg),)c.1666G>A p.Val556Ile), 1 mnp (c.1985_1986delGTinsAC p.Ser662Asn), 2 variant complex (c.1917_1921delGCCGGinsACCGT p.Ala641Ser), (c.1997_2001delACCTCinsGCCTG p.Asn666Ser)

169	1 SPN(c.10G>A p.Ala4Thr)	1 complex (c.2341_2343delGCCinsACT p.Ala781Thr)	2 SNP (c.1666G>A p.Val556Ile), (c.1921G>T p.Ala641Ser), 2 MNP(c.1031_1032delAGinsGA p.Lys344Arg), (c.1985_1986delGTinsAC p.Ser662Asn), 1 complex (c.1997_2001delACCTCinsGCCTG p.Asn666Ser)
176	1 SNP (c.1284G>T p.Glu428Asp)	0	1 SNP (c.1970G>C p.Gly657Ala)
181	0	0	0
182	0	0	1 MNP(c.1985_1986delGTinsAC p.Ser662Asn), 1 complex (c.1997_2001delACCTCinsGCCTG p.Asn666Ser)
223	0	1 SNP (c.559G>T p.Ala187Ser)	1 MNP (c.1985_1986delGTinsAC p.Ser662Asn), 1 complex (c.1997_2001delACCTCinsGCCTG p.Asn666Ser)
224	1 SNP (c.1276G>A p.Ala426Thr)	0	1 MNP (c.1985_1986delGTinsAC p.Ser662Asn), 1 complex (c.1997_2001delACCTCinsGCCTG p.Asn666Ser)
225	0	0	2 SNP (c.1666G>A p.Val556Ile), (c.1921G>T p.Ala641Ser), 2MNP (c.1031_1032delAGinsGA p.Lys344Arg), (c.1985_1986delGTinsAC p.Ser662Asn), 1 complex (c.1997_2001delACCTCinsGCCTG p.Asn666Ser)

227	0	0	2 SNP (c.1666G>A p.Val556Ile), (c.1921G>T p.Ala641Ser), 2 MNP (c.1031_1032delAGinsGA p.Lys344Arg) (c.1985_1986delGTinsAC p.Ser662Asn), 1 complex(c.1997_2001delACCTCinsGCCTG p.Asn666Ser)
233	0	0	1 MNP (c.1985_1986delGTinsAC p.Ser662Asn), 1 complex (c.1997_2001delACCTCinsGCCTG p.Asn666Ser)
235	0	0	0
188	0	0	1 MNP (c.1985_1986delGTinsAC p.Ser662Asn), 1 complex (c.1997_2001delACCTCinsGCCTG p.Asn666Ser), 1 complex (c.1997_2001delACCTCinsGCCTG p.Asn666Ser)
189	1 SNP (c.265A>G p.Lys89Glu)	0	1 MNP (c.1985_1986delGTinsAC p.Ser662Asn), 1 complex (c.1997_2001delACCTCinsGCCTG p.Asn666Ser)
193	0	0	0

198	2 SNP (c.1172C>T p.Ala391Val), (c.10G>A p.Ala4Thr)	0	1 SNP (c.1666G>A p.Val556Ile), 3 complex (c.1917_1921delGCCGGinsACCGT p.Ala641Ser), (c.1985_1986delGTinsAC p.Ser662Asn), (c.1997_2001delACCTCinsGCCTG p.Asn666Ser)
202	1 SNP (c.1172C>T p.Ala391Val)	1 complex (c.2341_2343delGCCinsACT p.Ala781Thr)	1 SNP (c.1666G>A p.Val556Ile), 2 MNP (c.1031_1032delAGinsGA p.Lys344Arg), (c.1985_1986delGTinsAC p.Ser662Asn), 2 complexes (c.1917_1921delGCCGGinsACCGT p.Ala641Ser), (c.1997_2001delACCTCinsGCCTG p.Asn666Ser)
206	1 MPN (c.5_6delGTinsAC p.Ser2Asn)	1 complex (c.837_841delCCTCAinsTCTCG p.Ser281Gly)	0
216	0	0	0
217	2 SNPS (.1172C>T p.Ala391Val), (c.10G>A p.Ala4Thr)	1 complex (c.2341_2343delGCCinsACT p.Ala781Thr)	1 SNP (c.1249G>A p.Ala417Thr), 2 MNP (c.1031_1032delAGinsGA p.Lys344Arg), (c.1985_1986delGTinsAC p.Ser662Asn), 1 complex (c.1997_2001delACCTCinsGCCTG p.Asn666Ser)
218			

219	2 SNP (c.1172C>T p.Ala391Val), (c.10G>A p.Ala4Thr)	0	1 SNP (c.1666G>A p.Val556Ile), 1 MNP (c.1985_1986delGTinsAC p.Ser662Asn), 2 complex (c.1917_1921delGCCGGinsACCGT p.Ala641Ser), (c.1997_2001delACCTCinsGCCTG p.Asn666Ser)
220	1 SNP (c.1172C>T p.Ala391Val)	1 complex (c.2341_2343delGCCinsACT p.Ala781Thr)	1 SNP (c.1666G>A p.Val556Ile), 2 MNP (c.1031_1032delAGinsGA p.Lys344Arg), (c.1985_1986delGTinsAC p.Ser662Asn), 2 complexes (c.1917_1921delGCCGGinsACCGT p.Ala641Ser), (c.1985_1986delGTinsAC p.Ser662Asn), 2 complex (c.1917_1921delGCCGGinsACCGT), (c.1997_2001delACCTCinsGCCTG p.Asn666Ser) p.Ala641Ser), (
221	1 SNP (c.1172C>T p.Ala391Val)	1 complex (c.2341_2343delGCCinsACT p.Ala781Thr)	1 SNP (c.1666G>A p.Val556Ile), 2 MNP (c.1031_1032delAGinsGA p.Lys344Arg), (c.1985_1986delGTinsAC p.Ser662Asn), 2 complex (c.1917_1921delGCCGGinsACCGT p.Ala641Ser), (c.1997_2001delACCTCinsGCCTG p.Asn666Ser)

Supplementary table S4. Genomics features of *P. aeruginosa* isolates.

***P. aeruginosa* isolates**	**Accession number**	**Source**	**Genome size (bp)**	**GC %**	**Contigs**	**Core genes**	**Shell genes**	**Pan/total genes**
PA17	NZ_QDGR00000000.1	Eye isolate	6360407	66.4	58	5524	545	6069
PA40	NZ_QDGW00000000.1	Eye isolate	6281273	66.4	97	5465	532	5997
PA55	NZ_QDGH00000000.1	Cystic fibrosis	6235426	66.6	76	5682	66	5748
PA57	NZ_QDGS00000000.1	Cystic fibrosis	6332663	66.5	70	5278	999	6277
PA59	NZ_CP024630.1	Cystic fibrosis	6289631	66.5	76	5485	561	6046
PA64	NZ_QDGF00000000.1	Cystic fibrosis	6264146	66.6	85	5470	533	6003
PA66	NZ_QDGE00000000.1	Cystic fibrosis	6337182	66.5	92	5478	637	6115
PA82	NZ_QDGM00000000.1	Eye isolate	6386862	66.5	60	5452	667	6119
PA86	NZ_QDGD00000000.1	Cystic fibrosis	6170765	66.5	75	5346	758	6104
PA92	NZ_QDGC00000000.1	Cystic fibrosis	6144291	66.6	79	5424	497	5921
PA100	NZ_QDGV00000000.1	Cystic fibrosis	6309141	66.5	78	5435	623	6058
PA102	NZ_QDGU00000000.1	Cystic fibrosis	6245346	66.5	61	5493	489	5982
PA149	NZ_QDGL00000000.1	Eye isolate	6314561	66.5	57	5491	526	6017
PA157	NZ_QDGK00000000.1	Eye isolate	6248837	66.5	51	5456	560	6016

PA171	NZ_QDGJ00000000.1	Eye isolate	6339042	66.5	58	5498	582	6080
PA175	NZ_QDGI00000000.1	Eye isolate	6757369	66.2	60	5446	1050	6496
PA121617	CP016214.1	Sputum	6430493	66.4	1	5497	589	6086
PA_D1	CP012585.1	Sputum	6643823	66.2	1	5437	924	6361
PA_D2	CP012578.1	Sputum	6642996	66.2	1	5435	924	6359
PA_D9	CP012580.1	Sputum	6645477	66.2	1	5435	935	6370
PA_D16	CP012581.1	Sputum	6681975	66.2	1	5437	961	6398
AAK/M5	NZ_JACGTC000000000.1	Environmental	6595587	65.3	155	5518	688	6206
AAK/M11	NZ_JACFOK000000000.1	Environmental	6737177	65.9	164	5486	935	6421
PAL1.16	NZ_JACEVD000000000.1	Pneumonia	6946945	65.9	130	5435	1270	6705
PAL1.34	NZ_JACEVA000000000.1	Pneumonia	6404462	66.5	40	5454	724	6178
PAL1.67	NZ_JACEUY000000000.1	Pneumonia	6926712	66.1	58	5468	1266	6734
PAL1.7	NZ_JACEQP000000000.1	Pneumonia	6625472	66.3	74	5437	899	6336
PAL1.5	NZ_JACEQR000000000.1	Pneumonia	7017998	66	63	5508	1243	6751
PAL1.8	NZ_JACEQO000000000.1	Pneumonia	6361965	66.5	38	5527	533	6060
NNPS180	NZ_JACCIH000000000.1	Soft tissue infection	6602334	66.2	140	5392	1023	6415
NNPS244	NZ_JACCII000000000.1	Soft tissue infection	6909183	66	141	5484	1175	6659
LESB58	NC_011770.1	Sputum	6601757	66.3	1	5493	831	6324
DK2	CP003149.1	Sputum	6402658	66.3	1	5386	851	6237
VRFPA04	CP008739.2	Corneal button	6818030	66.5	1	5137	1622	6759

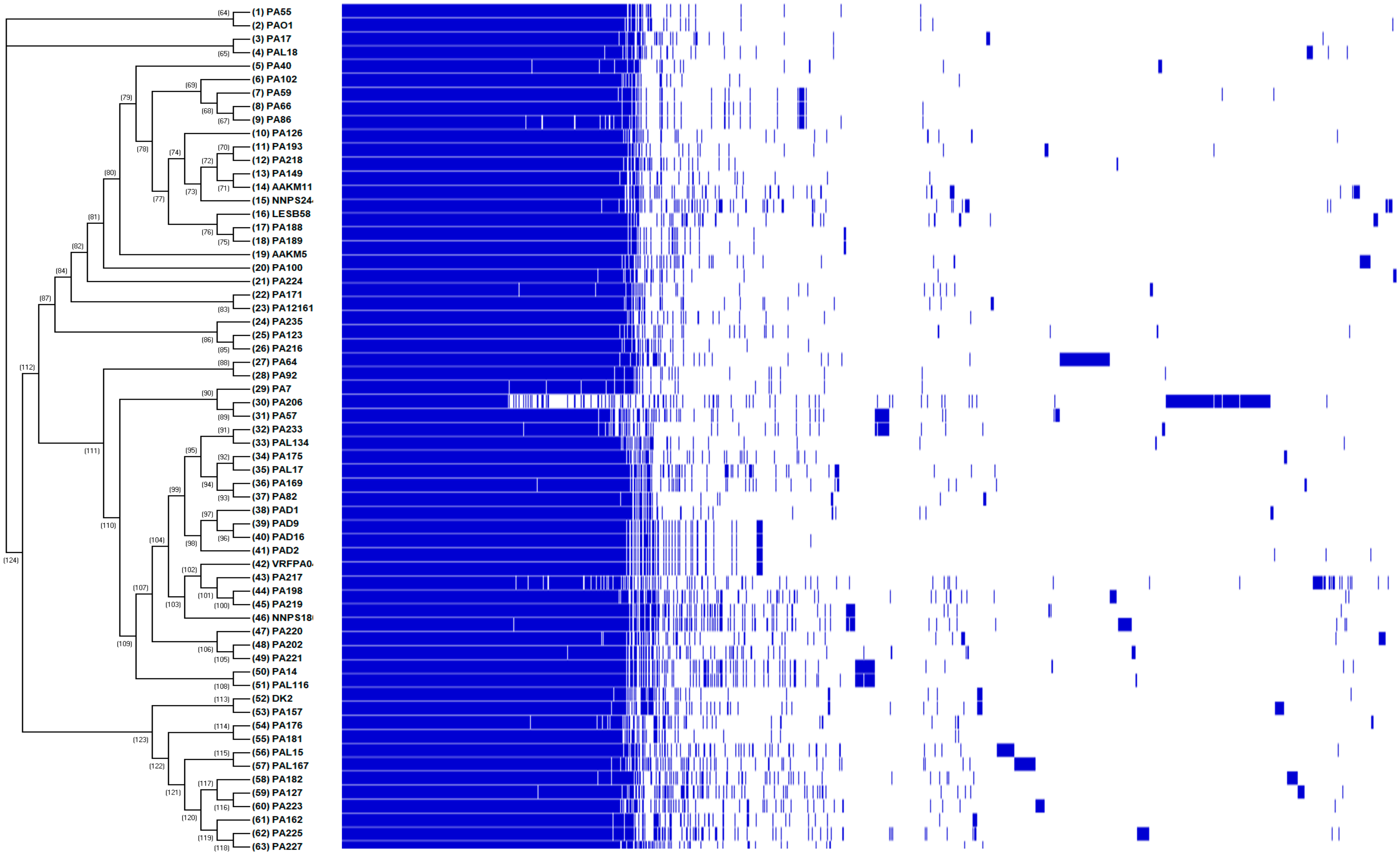
(1) PA55
(2) PAO1
(3) PA17
(4) PAL18
(5) PA40
(6) PA102
(7) PA59
(8) PA66
(9) PA86
(10) PA126
(11) PA193
(12) PA218
(13) PA149
(14) AAKM11
(15) NNPS24
(16) LESB58
(17) PA188
(18) PA189
(19) AAKM5
(20) PA100
(21) PA224
(22) PA171
(23) PA12161
(24) PA235
(25) PA123
(26) PA216
(27) PA64
(28) PA92
(29) PA7
(30) PA206
(31) PA57
(32) PA233
(33) PAL134
(34) PA175
(35) PAL17
(36) PA169
(37) PA82
(38) PAD1
(39) PAD9
(40) PAD16
(41) PAD2
(42) VRFPA0
(43) PA217
(44) PA198
(45) PA219
(46) NNPS18
(47) PA220
(48) PA202
(49) PA221
(50) PA14
(51) PAL116
(52) DK2
(53) PA157
(54) PA176
(55) PA181
(56) PAL15
(57) PAL167
(58) PA182
(59) PA127
(60) PA223
(61) PA162
(62) PA225
(63) PA227

Supplementary Figure S1. Pan-genome phylogenetic tree. The data on the right of the figure shows the presence and absence of genes. The tree was built using the genome of PAO1 as a reference.
